# *Candida tropicalis PMT2* Is a Dispensable Gene for Viability but Required for Proper Interaction with the Host

**DOI:** 10.3390/jof10070502

**Published:** 2024-07-20

**Authors:** Marco J. Hernández-Chávez, Iván Martínez-Duncker, Diana M. Clavijo-Giraldo, Luz A. López-Ramirez, Héctor M. Mora-Montes

**Affiliations:** 1 Departamento de Biología, División de Ciencias Naturales y Exactas, Campus Guanajuato, Universidad de Guanajuato, Noria Alta s/n, col. Noria Alta, C.P., Guanajuato 36050, GTO, Mexico; marco.hernandez@ipicyt.edu.mx (M.J.H.-C.); dm.clavijogiraldo@ugto.mx (D.M.C.-G.); adrianalr@ugto.mx (L.A.L.-R.); 2 Laboratorio de Glicobiología Humana y Diagnóstico Molecular, Centro de Investigación en Dinámica Celular, Instituto de Investigación en Ciencias Básicas y Aplicadas, Universidad Autónoma del Estado de Morelos, Cuernavaca 62209, MOR, Mexico; duncker@uaem.mx

**Keywords:** fungal cell wall, glycosylation, adhesion, *O*-linked mannan, phagocytosis, virulence, β-1,3-glucan, innate immune sensing

## Abstract

Candidemia is an opportunistic mycosis with high morbidity and mortality rates. Even though *Candida albicans* is the main causative agent, other *Candida* species, such as *Candida tropicalis*, are relevant etiological agents of candidiasis and candidemia. Compared with *C. albicans*, there is currently limited information about *C. tropicalis’* biological aspects, including those related to the cell wall and the interaction with the host. Currently, it is known that its cell wall contains *O*-linked mannans, and the contribution of these structures to cell fitness has previously been addressed using cells subjected to chemical treatments or in mutants where *O*-linked mannans and other wall components are affected. Here, we generated a *C. tropicalis pmt2*∆ null mutant, which was affected in the first step of the *O*-linked mannosylation pathway. The null mutant was viable, contrasting with *C. albicans* where this gene is essential. The phenotypical characterization showed that *O*-linked mannans were required for filamentation; proper cell wall integrity and organization; biofilm formation; protein secretion; and adhesion to extracellular matrix components, in particular to fibronectin; and type I and type II collagen. When interacting with human innate immune cells, it was found that this cell wall structure is dispensable for cytokine production, but mutant cells were more phagocytosed by monocyte-derived macrophages. Furthermore, the null mutant cells showed virulence attenuation in *Galleria mellonella* larvae. Thus, *O*-linked mannans are minor components of the cell wall that are involved in different aspects of *C. tropicalis*’ biology.

## 1. Introduction

*Candida* spp. are yeast usually found as part of the microbiota of the gastrointestinal tract and urogenital system. In healthy populations, they are commensals but can transit to a pathogenic lifestyle, causing superficial infections of skin and nails, or even deep-seated mycosis in immunocompromised patients, such as systemic candidiasis [1]. Nosocomial bloodstream *Candida* infections are found worldwide and are a life-threatening disease with high mortality rates of up to 71% [2,3]. Even though *Candida albicans* is the most common etiological agent of systemic candidiasis, other *Candida* species are associated with the disease and represent a significant burden for health systems [3,4,5]. *Candida tropicalis* is part of the clinically relevant *Candida* species that affect immunocompromised patients. It is frequently isolated in tropical countries but distributed worldwide and is responsible for 33–48% of systemic candidiasis cases [6,7]. Moreover, the isolation frequency increased 1.4-fold in intensive care unit patients during the COVID-19 pandemic outbreak [8]. This is the most frequently isolated species in Pakistan and India [7], and the most common *Candida* species isolated from neutropenic patients [6].

One point in common among the medically relevant fungal species, except *Cryptococcus* spp., is that the cell wall is the outermost fungal structure, and consequently, the first in establishing interaction with the host cells and tissues [9,10,11,12]. Contrary to *C. albicans*, a thoroughly studied fungal species, the *C. tropicalis* cell wall has been poorly studied. It contains chitin, β-1,6- and β-1,3-glucans, and *N*-linked mannans [13,14,15]. This oligosaccharide is composed of a Man_9_GlcNAc_2_ *N*-linked mannan core modified with an α-1,6-polymannose outer chain, which contains lateral chains of α-1,2-mannose units [16]. These lateral chains may be further modified with β-1,2-mannose units or phosphomannan [16,17]. When compared to *C. albicans*, *C. tropicalis* has increased cell wall porosity but similar chitin, glucan, and mannan content [15]. Like other *Candida* species, the *C. tropicalis* cell wall contains *O*-linked mannans that are linear mannose-based oligosaccharides and are in similar abundance to those found in the *C. albicans* cell wall [15]. 

In *C. albicans*, both *N*-linked and *O*-linked mannans are relevant cell wall components for tissue adhesion, virulence, and sensing by host immunity [15,18,19,20,21,22,23,24,25,26,27,28,29,30,31,32,33,34]. Studies on the role of *C. tropicalis* cell wall components during interaction with the host are scarce. This organism induces a stronger cytokine response by human peripheral blood mononuclear cells (PBMCs) than *C. albicans* in a dectin-1-dependent pathway when fungal cells are heat-killed, indicating a strong contribution of β-1,3-glucan sensing for cytokine stimulation [15]. Moreover, the phagocytosis of *C. tropicalis* by human monocyte-derived macrophages is higher than in *C. albicans* cells, involving a phosphomannan-dependent pathway [15]. Different from *C. albicans*, *C. tropicalis* induces fungipod formations in dendritic cells [17,35]. Mincle, a pattern recognition receptor that binds α-mannans contained in cell wall mannans, is dispensable for controlling systemic *C. albicans* infections but plays a role during systemic candidiasis caused by *C. tropicalis* [34]. In addition, it has been established that *C. tropicalis O*-linked mannans are dispensable for cytokine production by human PBMCs, but *N*-linked mannans and β1,3-glucan are essential to stimulate cytokine production via co-stimulation of both dectin-1 and mannose receptor [17], and *N*-linked mannans are required for proper biofilm formation in *C. tropicalis* [36].

Protein-*O*-mannosyltransferases (Pmts) are endoplasmic reticulum (ER) membrane-bound proteins that transfer mannose residues to the lateral chain of serine/threonine residues in a co- or posttranslational mechanism [37]. This enzyme activity starts the *O*-linked mannosylation pathway, which continues in the Golgi complex, where members of the *MNT* gene family extend the *O*-linked mannan to up to seven α-1,2-mannose units [23,38]. In *C. albicans*, five Pmts have been identified: Pmt1 and Pmt5 (belonging to the Pmt1 subfamily), Pmt2 and Pmt6 (members of the Pmt2 subfamily), and Pmt4 [39]. Currently, null mutants for *PMT* genes have been generated, except *PMT2*, which is essential and cannot be complemented by *PMT6*, the other subfamily member [39]. The heterozygous *PMT2*/*pmt2*∆ strain was incapable of growing at 42 °C, was sensitive to high salt concentrations, and showed increased sensitivity to cell wall-perturbing agents and antifungal drugs [39]. Thus, it has been proposed that Pmt2, along with Pmt1 and Pmt4 are responsible for most of the Pmt activity, and Pmt5 and Pmt6 have a regulatory activity, in particular of the targeted protein for *O*-linked mannosylation [37]. Similar to *Saccharomyces cerevisiae*, it has been proposed that Pmt1 and Pmt2 form a functional heterodimer to catalyze the transference of the monosaccharide to proteins [40].

Currently, there is limited information on the contribution of *O*-linked mannans to *C. tropicalis* biology and interaction with the host. The approaches used to study this cell component are the chemical removal by β-elimination or the analysis of the *pmr1*∆ null mutant, which is affected in both *N*-linked and *O*-linked mannan synthesis [15,17]. *PMR1* product is a Golgi-resident P-type Ca^2+^/Mn^2+^-ATPase that controls Mn^2+^ homeostasis in this organelle and consequently regulates the Mn^2+^-dependent mannosyltransferases [20]. Here, we generated a *C. tropicalis pmt2*∆ null mutant and performed phenotypical characterization, in particular on the cell wall composition and the interaction with the host.

## 2. Materials and Methods

### 2.1. Strains and Culturing Conditions

The strains used in this study are listed in Table 1. Yeast maintenance and propagation were performed by growing at 28 °C in YPD medium [2% (*w*/*v*) bacteriological peptone, 1% (*w*/*v*) yeast extract, and 2% (*w*/*v*) dextrose] or SC medium [0.67% (*w*/*v*) yeast nitrogen base with ammonium sulfate with amino acids, and 2% (*w*/*v*) glucose]. When a solid medium was required, the broth was added with 2% (*w*/*v*) agar. Filamentation in the solid medium was stimulated by growing yeast cells on solid Spider medium at 30 °C [41], while in a liquid medium, it was stimulated in RPMI 1640 supplemented with 2.5% (*v*/*v*) fetal calf serum and incubating 3.5 h at 37 °C and shaking (150 rpm) [42]. For recycling of the *SAT1* marker, cells were grown in YEP broth [2% (*w*/*v*) bacteriological peptone and 1% (*w*/*v*) yeast extract] at 28 °C. When required, cells were heat-killed by incubating at 56 °C for 60 min [43]. For analysis of the *C. tropicalis*–host interaction, yeasts were grown at 28 °C in YPD broth until reaching the logarithmic growth phase. When required, cells were heat-inactivated at 56 °C for 60 min. The viability lost was confirmed on YPD plates incubated at 28 °C for 48 h. For β-elimination, cells were suspended in 10 mL of NaOH 0.1 N and incubated at room temperature during 18 h, as reported [17]. The preparations were neutralized with HCl 0.1 N, and the cells pelleted by centrifuging, suspended in sterile phosphate-buffered saline (PBS), and kept at −20 °C until used. 

### 2.2. Complementation of Candida albicans Mutants

To generate the complementation plasmid, the open reading frame (ORF) of CTRG_05668 (https://www.ncbi.nlm.nih.gov/nuccore/NW_003020061.1?from=229097&to=231394&report=fasta, accessed on 18 January 2019) was amplified by PCR with the primer pair 5’-AAGCTTATGTCTACTTCTGTTGAACCA-3’ and 5’-GCTAGC CTAGGTATATTTATCATCAGA-3’ (underlined sequences are added restriction sites for HindIII and NheI, respectively, to direct ligation to the plasmid). The 2298 bp amplicon was cloned into pCR^®^2.1-TOPO^®^ (Thermo Fisher Scientific, Waltham, MA, USA), and then subcloned into the HindIII and NheI sites of pACT1-GFP [44], generating pACT1-CTRG_05668, which was digested with StuI and used to transform a *C. albicans PMT2*/*pmt*2∆ heterozygous mutant and a *pmt6*∆ null mutant [39,45]. Plasmid integration into the neutral *RPS10* locus was confirmed by PCR, as reported [46]. 

The analysis of cell growth in the presence of cell wall-perturbing agents was performed as previously reported [17]. YPD-grown cells were washed three times with PBS, cell concentration adjusted at an OD_600nm_ = 1.0, and diluted to achieve an OD_600nm_ = 0.01 with fresh medium. Aliquots of 95 µL were added into 96-well plates, 5 µL of compounds were added to each well, plates were incubated 24 h at 28 °C and the absorbance at 600 nm was measured. Calcofluor white and Congo red were from Sigma-Aldrich (San Louis, MO, USA), and hygromycin B was from GoldBio (St Louis, MO, USA). Control wells containing 5 μL of PBS instead of the tested compounds were used to assess normal growth and to normalize data obtained with the cell wall-perturbing agents. Data are shown as a percentage of fungal growth without perturbing compounds.

**Table 1:** Strains used in this work.

Strain	Organisms	Origin	Genotype	Reference
NGY152	*C. albicans*	Derived from CAI-4	*ura3*Δ*-iro1*Δ::*imm434*/*ura3*Δ-*iro1*Δ::*imm434*; *RPS1*/*rps1*∆::CIp10	[46]
P2-67	*C. albicans*	Derived from CAI-4	*ura3*Δ*-iro1*Δ::*imm434*/*ura3*Δ-*iro1*Δ::*imm434*; *PMT2*/*pmt2*Δ::*hisG*	[39]
CAP2-2341	*C. albicans*	Derived from CAI-4	*ura3*Δ*-iro1*Δ::*imm434*/*ura3*Δ-*iro1*Δ::*imm434*; *pmt6*Δ::*hisG*/*pmt6*Δ::*hisG*	[45]
HMY211	*C. albicans*	Derived from P2-67	As P2-67 but *RPS1*/*rps1*∆::CIp10	This work
HMY212	*C. albicans*	Derived from P2-67	As P2-67 but *RPS1*/*rps1*∆:: pACT1-CTRG_05668	This work
HMY213	*C. albicans*	Derived from CAP2-2341	As CAP2-2341 but *RPS1*/*rps1*∆::CIp10	This work
HMY214	*C. albicans*	Derived from CAP2-2341	As CAP2-2341 but *RPS1*/*rps1*∆:: pACT1-CTRG_05668	This work
ATCC^®^ MYA-3404	*C. tropicalis*	ATCC	Wild type	ATCC
HMY215	*C. tropicalis*	MYA-3404	As ATCC MYA-3404, but *pmt2*Δ::*sat1*/*PMT2*	This work
HMY216	*C. tropicalis*	HMY215	As ATCC MYA-3404, but *pmt2*Δ/*PMT2*	This work
HMY217	*C. tropicalis*	HMY216	As ATCC MYA-3404, but *pmt2*Δ/*pmt2*Δ::*sat1*	This work
HMY218	*C. tropicalis*	HMY217	As ATCC MYA-3404, but *pmt2*Δ/*pmt2*Δ	This work
HMY219	*C. tropicalis*	HMY218	As ATCC MYA-3404, but *pmt2*Δ/*pmt2*Δ::*sat1-PMT2*	This work
HMY220	*C. tropicalis*	HMY219	As ATCC MYA-3404, but *pmt2*Δ/*pmt2*Δ::*PMT2v*	This work

### 2.3. Disruption of Candida tropicalis PMT2

The *SAT1* flipper method was used to disrupt the two *PMT2* alleles [17]. To generate homologous recombination arms, a fragment of 1368 bp upstream the CTRG_05668 ORF was amplified by PCR, using the primer pair 5’-GGGCCCAGCTGCAAAATTAGTCTTT-3’ and 5’-CTCGAGTTGCTTGTTGTTTGTTGCTT-3’(underlined sequences correspond to added restriction sites for cloning); cloned in the pCR^®^2.1-TOPO^®^ (Thermo Fisher Scientific); and then subcloned into ApaI and XhoI sites of pSFS2, generating pSFS2-upstream [47]. A similar strategy was used to generate the downstream recombination arm. For this, a 1201-bp amplicon was generated with the primer pair 5’-GCGGCCGCGACATTTCGCCTTTGATCCAAC-3’ and 5’-GAGCTCGTTGACTTGCCAATAAAAGAAGAAG-3’ (underlined sequences correspond to added restriction sites for cloning) and cloned into the NotI and SacI sites of pSFS2-upstream, generating the complete disruption cassette, which was ApaI- and SacI-digested before the fungal transformation. Strain ATCC^®^ MYA-3404, considered as the wild-type (WT) parental strain here, was transformed with the disruption cassette and transformants were selected on YPD plates added with 200 µg mL^−1^ nourseothricin (Goldbio). To recycle the *SAT1* marker, cells were grown for 2 days at 28 °C in YEP broth added with 2% (*w*/*v*) maltose and selected on YPD plates supplemented with 10 µg mL^−1^ nourseothricin. The strategy was repeated to disrupt the second allele. In all cases, PCR with primer pairs aligning inside and outside of the recombination regions was used to assess recombination events in the targeted locus.

To generate a reintegrant control strain, the CTRG_05668 ORF plus 1000 bp upstream and 500 bp downstream was amplified by PCR using the following primer pairs: 5’-GGGCCCGAGAGAGCAAACACATTAG-3’ and 5’-CTCGAGTTTAGGTATTATTCTTATATG-3’ (underlined sequences correspond to added restriction sites for cloning). The 3978 bp was cloned into the ApaI and XhoI sites of pSFS2 [17]. Then a 700 bp fragment downstream for the target ORF was amplified and cloned into the NotI and SacI sites of the same plasmid. This fragment was amplified with the primer pair 5’-GCGGCCGCATACCTAGATTTTATCTTCGCAT-3’ and 5’-GAGCTCATTAATCTAGTATATTTAAAATCATC-3’ (underlined sequences correspond to added restriction sites for cloning). The reintegration cassette was released by digesting with ApaI and SacI and used in fungal transformation. Similar to the null mutant strain generation, the *SAT1* was recycled to have WT, null mutant, and reintegrant control strains sensitive to nourseothricin.

### 2.4. Gene Expression Analysis

Total RNA was extracted from yeasts and used for cDNA synthesis with an oligo(dT)20 primer. The cDNA was purified by chromatography and quantified in a NanoDrop 2000 (Thermo Fisher Scientific). Quantitative PCR reactions contained cDNA, SYBR Green PCR Master Mix, and a specific primer pair, and were performed in a thermocycler StepOne Plus (Life Technologies, Carlsbad, CA, USA). For data normalization, the *ACT1* gene was amplified [48]. Data were analyzed in the StepOne software V 2.2 (Life Technologies) using the 2^−∆∆Ct^ method [49]. The following primer pairs were used: for *ACT1*, 5’-GACCGAAGCTCCAATGAATC-3’ and 5’-AATTGGGACAACGTGGGTAA-3’; for *PMT1*, 5’-ACTTCTCATCACCACTGGCA-3’ and 5’-CGGCTGCCTAAGTAACCAAC-3’; for *PMT2*, 5’-GTGGTCCACGTGAAGTTTCC-3’ and 5’-TGGTGCTTGAACATCATGGG-3’; for *PMT4*, 5’-TGGTCCAGGTGATGCTTTCA-3’and 5’-TTTACCAGCACCTTCAACGG-3’; for *PMT5*, 5’-TCCGAAATTTTCATTCTGGCAA-3’ and 5’-CTGGTGAGTCAGTTGGTTGT-3’; for *PMT6*, 5’-AACTGCCGTGCTCTTAGGAT-3’ and 5’-GCAGTGACAAAGAGACCCAC-3’; for *PKC1* (CTRG_02299), 5’-CCGGTGGTGATTTGATGTGG-3’ and 5’-CAAATGCCCACCAGTCAACA-3’; and for *MKC1* (CTRG_01071), 5’-CGATGGCACGTCAACTAAGG-3’ and 5’-TTCCCGGTTTCAAGTCTCGA-3’.

### 2.5. Analysis of the Cell Wall

Yeasts were grown overnight at 28 °C in YPD, harvested by centrifuging, washed three times with PBS, and disrupted with glass beads in an MSK cell homogenizer (Braun, Melsungen, Germany). Homogenates were centrifuged, pelleted cell walls were saved, washed three times with PBS, and cleaned with hot SDS, β-mercaptoethanol, and NaCl, as reported [22]. Five mg of the cell wall were hydrolyzed with 2 M trifluoroacetic acid; the acid was removed by evaporation, and samples were analyzed by high-performance anion-exchange chromatography with pulsed amperometric detection (HPAEC-PAD), as reported [50]. For this purpose, a CarboPac PA200 analytical column (3 × 250 mm) with a CarboPac PA200 guard column (3 × 50 mm) was used. Samples were eluted with an isocratic gradient of 3.2 mM NaOH with a flux rate of 0.15 mL min^−1^ for 20 min. For protein quantification, walls were boiled with 1 N NaOH for 30 min, and after neutralization with 1 N HCl, the protein content was quantified with the Pierce BCA Protein Assay (Thermo Fisher Scientific) [22].

To quantify *N*-linked mannans, cells were adjusted to 1 × 10^9^ cells mL^−1^, suspended in 3 mM NaOAc and 25 U endoglycosidase H (New England Biolabs, Ipswich, MA, USA), and incubated at 37 °C for 24 h [51]. Reactions were neutralized, and centrifuged, and the soluble mannans were saved and kept at −20 °C until use. For *O*-linked mannans, a similar approach was used but cells were incubated overnight at room temperature with 0.1 N NaOH under gentle orbital shaking [38]. In both cases, mannans were hydrolyzed with trifluoroacetic acid and analyzed by HPAEC-PAD, as described [51]. The chitin exposure at the cell wall surface was analyzed by incubating yeasts with 0.5 mg mL^−1^ fluorescein isothiocyanate-conjugated wheat germ agglutinin (Sigma-Aldrich) for 60 min at room temperature [52]. For β-1,3-glucan analysis, yeasts were incubated with 5 μg mL^−1^ IgG Fc-dectin-1 chimera [53] for 40 min at room temperature followed by an incubation step with 1 μg mL^−1^ donkey anti-Fc IgG-FITC (Sigma-Aldrich). Fluorescence associated with cells was observed with a Zeiss Axioscope-40 microscope (Carl Zeiss AG, Jena, Germany) and an Axiocam MRc camera. A total of 300 cells were analyzed per sample, and the median fluorescence per cell was calculated, as described [54]. 

Cell wall porosity was analyzed by the relative ability of polycations to interact with the plasma membrane, as described [55], while phosphomannan content was estimated by the cellular ability to bind Alcian blue, as previously reported [56].

### 2.6. Analysis of Enzyme Activity

Secreted proteolytic activity was assessed by the degradation of bovine serum albumin (BSA) as reported. [57]. Overnight-grown yeast cells in YPD were washed three times with PBS, pelleted by centrifuging, with cell concentration adjusted to OD_600nm_ = 0.4, and 100 µL was mixed with 300 µL 5.0% (*w*/*v*) BSA (Sigma-Aldrich) in 50 mM sodium citrate, pH 3.2, and incubated for 30 min at 37 °C. Reactions were centrifuged, the supernatant collected, 100 µL 2 M perchloric acid was added, and the mixture was incubated for 15 min at 4 °C. Protein content was estimated by reading the absorbance at 280 nm. Mock reactions with only cells supernatant were used to establish basal absorbance at 280 nm. The change in absorbance between the test and mock reactions per minute was defined as one enzyme unit. 

For secreted lipolytic activity, aliquots of 100 µL cells adjusted to OD_600nm_ = 0.4 were added with 100 µL of 40 mM 4-methylumbelliferyl palmitate (Sigma-Aldrich) in 50 mM MES-Tris buffer, pH 6.0, and incubated for 30 min at 37 °C. Then, 200 µL 50 mM glycine-NaOH buffer, pH 11.0, was added, reactions were centrifuged, and the supernatants were used to quantify the released 4-methylumbelliferone in an LS-5B luminescence spectrofluorometer (Perkin-Elmer, Waltham, MA, USA). Excitation and emission wavelengths were set at 350 nm and 440 nm, respectively, as reported [58]. One enzyme unit was defined as one nmole 4-methylumbelliferone generated per minute.

To determine intracellular enzyme activities, cells were pelleted by centrifuging and disrupted in an MSK cell homogenizer (Braun). Cell homogenates were treated as described above.

### 2.7. Sensitivity to Antifungal Drugs

Overnight cultures were used to inoculate fresh YPD broth and incubated at 28 °C until the exponential phase (typically 5 h). Cells were harvested by centrifuging, washed three times with PBS, and suspended in RPMI-1640 medium with MOPS and L-glutamine, without sodium bicarbonate (Sigma-Aldrich). The sensitivity to antifungal drugs was assessed following the M27-A3 protocol, while the interpretation of minimal inhibitory concentrations (MIC) was performed as suggested in the M27-S4 protocol [59]. The drugs tested were amphotericin B, fluconazole, and caspofungin (all from Sigma-Aldrich). Amphotericin B and caspofungin were tested in a range from 32 to 0.125 μg mL^−1^, whilst for fluconazole, the dilutions tested were from 1024 to 1 μg mL^−1^.

### 2.8. Analysis of Biofilm Formation

Biofilm stimulation and analysis were performed as reported [60,61]. Yeasts were suspended in PBS, cell concentration adjusted to 1 *×* 10^7^ cells mL*^−^*^1^, and 100 µL PBS was placed in flat-bottom Nunc polystyrene 96-microtiter plates (Thermo Fisher Scientific). Cell adhesion was allowed to occur for 4 h at 30 °C. Then, wells were washed three times with PBS, 100 µL RPMI-1640 medium supplemented with L-glutamine (Sigma-Aldrich) was added to each well, and plates were incubated for 24 h at 37 °C. Cell biomass within biofilms was assessed by staining with 0.02% (*w*/*v*) crystal violet and reading absorbance at 590 nm, as reported [60]. To analyze the biofilm extracellular matrix, the media were removed from mature biofilms, and 200 µL of 50 µg mL*^−^*^1^ chitinase from Streptomyces griseus (Sigma-Aldrich) was added to each well and incubated at 25 °C for 2 h. Then, wells were sonicated for 5 min with a Q700 Sonicator (Thomas Scientific, Swedesboro, NJ, USA), and the supernatant was collected after centrifuging and kept at −20 °C until used [62]. These soluble fractions were concentrated in an Amicon Ultra centrifugal filter with Ultracel-3K (Sigma-Aldrich) and used to quantify glucose and glucosamine by HPAEC-PAD, as reported [51]. The pelleted cells from detached biofilms were serially diluted and grown on YPD plates at 28 °C for 24 h, for colony-forming unit quantification.

### 2.9. Adhesion Assays

Adhesion to components of the extracellular matrix was analyzed by ELISA [63]. Plates were prepared by adding 100 µL of 1.0 µg 100 µL^−1^ of extracellular matrix components in 0.05% (*w*/*v*) PBS–Tween 20 to wells of Nunc MaxiSorp™ flat-bottom 96-well microplates (Thermo Fisher Scientific). These were incubated for 3 h at room temperature, washed three times with 0.05% (*w*/*v*) PBS–Tween 20, and blocked with 1% (*w*/*v*) bovine serum albumin (BSA) in PBS overnight at 4 °C. Plates were washed three times with 0.05% (*w*/*v*) PBS–Tween 20 before assessing adhesion.

Aliquots of 100 µL containing 5 × 10^6^ yeasts in PBS were added per well, and incubated for 1 h at 37 °C. Then, plates were washed three times with 0.05% (*w*/*v*) PBS–Tween 20, 100 µL of polyclonal anti-*Candida* antibody (Thermo-Fisher Scientific, cat number PA1-27158) diluted at 1:3000 was added, and the plates were incubated for 2 h at room temperature. Plates were washed with 0.05% (*w*/*v*) PBS–Tween 20, 100 µL of goat anti-rabbit IgG-peroxidase antibody (Sigma-Aldrich) was added at a working dilution of 1:5000, and the plates were further incubated for 2 h at room temperature. Peroxidase activity was revealed with 0.1 mg mL^−1^ 2,2′-azino-bis(3-ethylbenzothiazoline-6-sulfonic acid) diammonium salt and 0.006% (*v*/*v*) hydrogen peroxide. Plates were incubated for 20 min at room temperature, 2 N sulfuric acid was added, and the plates were read at 450 nm in a Varioskan LUX Multimode Microplate Reader (Thermo Fisher Scientific). The extracellular matrix components were human laminin, elastin, fibrinogen, recombinant fibronectin, recombinant thrombospondin-1, type I collagen, or bovine type II collagen (all from Sigma-Aldrich).

### 2.10. Ethics Statement

This study involved the use of human blood. Samples were withdrawn from healthy donors who previously signed an informed consent statement. This study was approved by the Ethics Committee of Universidad de Guanajuato (reference CIBIUG-P26-2020) and conducted following the Declaration of Helsinki.

### 2.11. Cytokine Stimulation by Human Peripheral Blood Mononuclear Cells

Human venous blood samples were collected in tubes containing EDTA, and used to separate PBMCs by differential centrifugation in Histopaque-1077 (Sigma-Aldrich), as reported [64]. The PBMC–*Candida* interactions were performed in U-bottom 96-well microplates, containing 200 µL RPMI 1640 Dutch modification (added with 2 mM glutamine, 0.1 mM pyruvate, and 0.05 mg mL^−1^ gentamycin; all reagents from Sigma-Aldrich), 1 × 10^5^ yeast-like cells, and 5 × 10^5^ PBMCs. Plates were incubated at 37 °C for 24 h with 5% (*v*/*v*) CO_2_ and centrifuged for 10 min at 1800× *g* at 4 °C. The supernatants were collected and kept at −20 °C until used for cytokine quantification by ELISA [17]. Human tumor necrosis factor-alpha (TNFα), interleukin 6 (IL-6), interleukin 1β (IL-1β), and interleukin 10 (IL-10) were quantified with Standard ABTS ELISA Development kits (Peprotech, Cranbury, NJ, USA). A calibration curve was included in each ELISA plate along with negative control wells, containing supernatant from PBMCs incubated only with culturing medium. In some experiments, human PBMCs were preincubated for 1 h at 37 °C with 200 μg mL^−1^ laminarin (Sigma-Aldrich) [17] before interacting with yeasts.

### 2.12. Phagocytosis Assays

Human PBMCs were differentiated into macrophages by incubating with recombinant human granulocyte–macrophage colony-stimulating factor (Sigma-Aldrich), as reported [54]. Yeasts were labeled with 1 mg mL^−1^ Acridine Orange (Sigma-Aldrich) at room temperature for 30 min, the excess dye was removed by washing with cold PBS, and cell concentration was adjusted to 3 × 10^7^ cells mL^−1^ [17]. The phagocytosis assays were performed in 6-well plates, with an immune cell/fungus ratio of 1:6 in 800 µL of DMEM medium (Sigma-Aldrich). The plates were incubated at 37 °C for 2 h and 5% (*v*/*v*) CO_2_ and cells were detached with cold PBS and incubated with 1.25 mg mL^−1^ Trypan Blue [17]. Cells were analyzed in a FACSCanto II system (Becton Dickinson, Franklin Lakes, NJ, USA) using the FL1 and FL2 channels calibrated with non-stained monocyte-derived macrophages. Fifty thousand events were collected per sample. The use of two channels allowed to classification of cells in the early, intermediate, and late stages of phagocytosis, as reported [65]. The preincubation with laminarin was performed as described in Section 2.11. 

### 2.13. Analysis of Virulence

The analysis was performed in *Galleria mellonella* larvae from an in-house colony [66]. Insects were fed ad libitum with a conventional diet based on corn bran and honey [67]. Only larvae measuring between 1.2 and 1.5 cm in length, with active behavior and no body melanization were included in this study. Cells were used at a concentration of 2 × 10^7^ yeasts in 10 μL of PBS to inoculate larvae in the last left pro-leg, using a Hamilton syringe and a 26-gauge needle, as reported [66]. Infected larvae were kept at 37 °C under hydration ad libitum with chopped apple. Insects were inspected daily for 15 days, and those without response to external stimuli and with extensive body melanization were considered dead [66]. A total of 30 animals was used for each strain. One additional group injected only with PBS was also included as a control. To quantify colony-forming units, hemocyte concentration, phenoloxidase activity, melanin production, and cytotoxicity, the hemolymph was collected, anticoagulated, and assayed as reported [67]. Phenoloxidase activity was assayed using 20 mM 3,4-dihydroxyDL-phenylalanine (Sigma-Aldrich), while cytotoxicity was quantified with the Pierce LDH Cytotoxicity Assay (Thermo Fisher Scientific) [67].

### 2.14. Statistical Analysis

GraphPad Prism 6 software, version 6.07 was used for data analysis. Ex vivo and in vivo experiments were analyzed with the Mann–Whitney U and Kruskal–Wallis tests, whereas other results were analyzed with the parametric Student’s test. Statistical significance was set at *p* < 0.05. Larva mortality is shown in Kaplan–Meier survival curves and the log-rank test was used for data analysis.

## 3. Results

### 3.1. Disruption of Candida tropicalis PMT2

**Figure 1:**
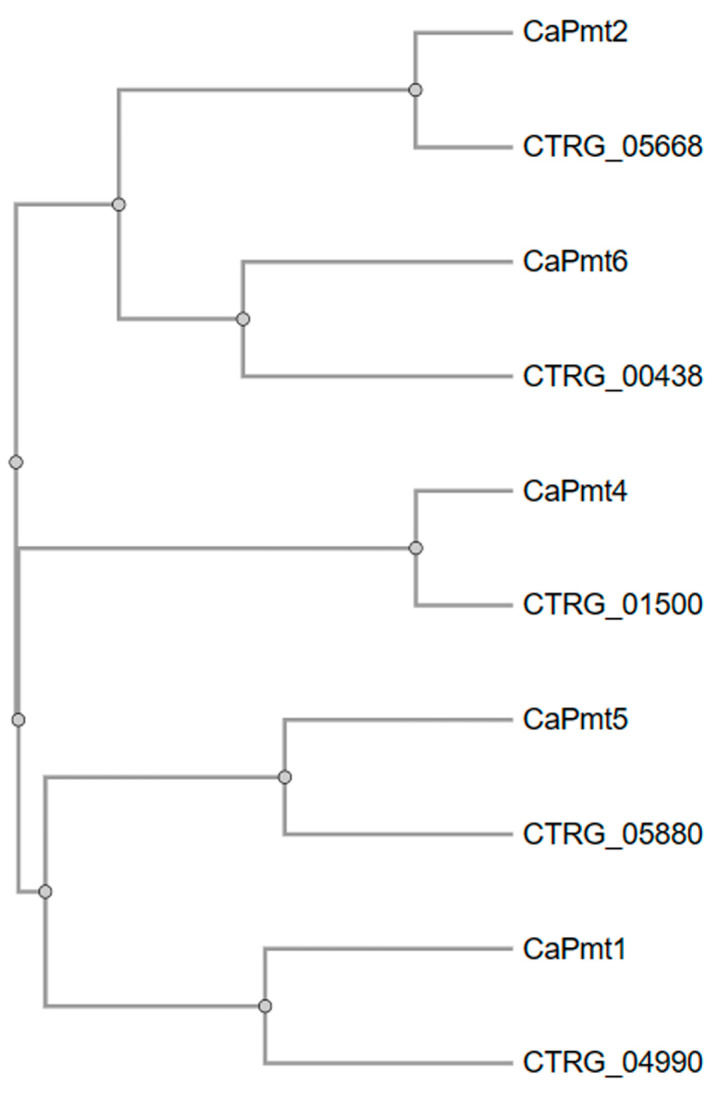
A guide tree constructed with *Candida albicans PMT* family members and putative orthologs on *Candida tropicalis*. The amino acid sequences of *C. albicans PMT* family members, along with the putative orthologs identified in *C. tropicalis*, were downloaded from (http://www.candidagenome.org/, accessed on 30 April 2024), used in multiple sequence alignments in Clustal Omega (https://www.ebi.ac.uk/jdispatcher/msa/clustalo, accessed on 30 April 2024), and from this, the guide tree was constructed. Labels starting with Ca are *C. albicans* sequences, whilst those beginning with CTRG are from *C. tropicalis*.

Thus far, the *C. tropicalis PMT* gene family has not been studied, and therefore, the putative functional orthologs in this species have been only predicted by bioinformatics analyses. Similar to *C. albicans*, there are five putative members of this gene family, and when comparative analyses were performed, it was predicted that *C. tropicalis* contains a putative ortholog for all of the *C. albicans PMT* family members (Figure 1). According to the guide tree constructed in Clustal Omega (https://www.ebi.ac.uk/jdispatcher/msa/clustalo, accessed on 30 April 2024), the sequence CTRG_05668 is the putative ortholog of *C. albicans PMT2* (Figure 1), and the putative protein showed a similarity of 91.4%, 61.7%, 54.3%, 46.9%, and 45.6% to *C. albicans* Pmt2, Pmt6, Pmt1, Pmt4, and Pmt5, respectively. The open reading frame encodes for a 765 amino acids protein, with seven putative transmembrane domains and the N-terminal predicted to be in the cytoplasm (https://services.healthtech.dtu.dk/services/TMHMM-2.0/, accessed on 30 April 2024), similar to *S. cerevisiae* Pmt2 [68]. 

**Figure 2:**
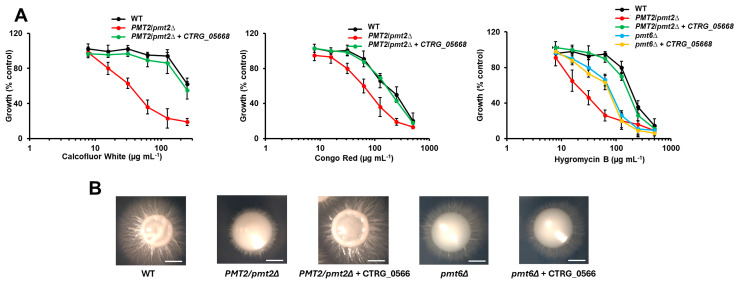
Complementation of *Candida albicans PMT2*/*pmt2*∆ heterozygous mutant with *Candida tropicalis* CTRG_05668. In (**A**), yeasts were incubated in YPD broth added with different concentrations of Congo red, calcofluor white, or hygromycin B and incubated for 24 h at 30 °C, and growth was measured by absorbance at 600 nm. Data are shown as a percentage of fungal growth in culture with no perturbing agent added. Data are means ± SD of three independent experiments performed in duplicates. The curves generated with the heterozygous mutant or the *pmt6*∆ null mutants were significantly different from those generated with the WT control strain or the heterozygous mutant complemented with CTRG_05668 (*p* < 0.05 when compared by two-way ANOVA). In (**B**), colony morphology after 5 days at 30 °C on Spider medium. Scale bars, 1 mm. Strains used are NGY152 (WT), HMY211 (*PMT2*/*pmt2*Δ), HMY212 (*PMT2*/*pmt2*Δ + CTRG_05668), HMY213 (*pmt6*Δ), and HMY214 (*pmt6*Δ + CTRG_05668).

Since Pmt2 and Pmt6 belong to the same subfamily, and the putative protein encoded by CTRG_05668 showed high similarity to both *C. albicans* Pmt2 and Pmt6, we expressed this gene in the *C. albicans PMT2*/*pmt2*∆ [39] and a *pmt6*∆ null mutant [45] to assess functional complementation. Since both mutant strains are Ura^--^, the heterologous complementation was performed with the pACT1-GFP, where the GFP ORF was substituted with the *C. tropicalis* CTRG_05668 ORF and transcription was controlled with the strong and constitutive *ACT1* promoter [44]. It was previously reported that the *C. albicans PMT2*/*pmt2*∆ heterozygous mutant showed increased sensitivity to the cell wall-perturbing agents calcofluor white, Congo red, and hygromycin B, along with poor filamentation in Spider medium and inability to grow at 42 °C [39]. After complementation with the CTRG_05668 ORF, the mutant strain restored the sensitivity to the three perturbing agents to levels similar to the WT control strain (Figure 2A) and underwent dimorphism in the solid Spider medium (Figure 2B). In addition, cells grew at 42 °C as the WT control strain. Contrary to the *PMT2* heterozygous mutant, the *pmt6*∆ null mutant has a more discrete phenotype, with increased sensitivity only to hygromycin B and delayed filamentation on solid medium [45]. Both phenotypes were not restored upon transformation with pACT1-CTRG_05668 (Figure 2A,B). Therefore, our result suggests that CTRG_05668 is the functional ortholog *C. albicans PMT2* and named hereafter *PMT2*.

**Figure 3:**
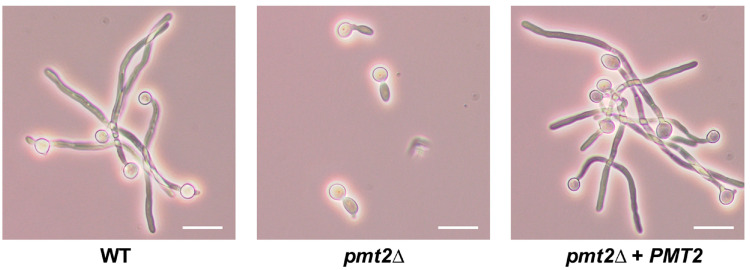
The *Candida tropicalis pmt2*∆ null mutant fails to undergo dimorphism. Yeast cells grown in RPMI 1640 supplemented with 2.5% (*v*/*v*) fetal calf serum for 3.5 h at 37 °C. Scale bars 10 µm. Strains used are MYA-3404 (WT), HMY218 (*pmt2*Δ), and HMY220 (*pmt2*Δ + *PMT2*).

A *C. tropicalis pmt2*∆ null mutant was generated following the *SAT1* flipper methodology and contrary to the observations in *C. albicans*, both alleles were disrupted with no viability loss. To associate any phenotype to the *PMT2* disruption, a reintegrant control strain was generated using the same recycling cassette. For both the null mutant and reintegrant control strain, the *SAT1* marker was recycled to generate nourseothricin-sensitive cells, like the WT control strain. In all cases, cassette integration into the targeted locus was confirmed by PCR The *pmt2*∆ did not form cell aggregates and the colony morphology was similar to that observed in the WT and reintegrant control strains. Despite this apparent normal phenotype, the *pmt2*∆ null mutant showed increased doubling times when compared with the WT control strain (doubling times of 1.52 ± 0.14 h and 2.28 ± 0.22 h, for WT and *pmt2*∆ null mutant, respectively, *p* < 0.05), and this was recovered to WT levels in the reintegrant strain (1.46 ± 0.28 h, *p* > 0.05 when compared with the WT control strain). The null mutant failed to grow at 42 °C and underwent dimorphism in both liquid and solid medium (Figure 3).

We hypothesized that *PMT2* disruption may affect the expression of other *PMT* family members, and thus, their expression was quantified in cells growing in YPD, SC medium, or under conditions to stimulate filament growth. In all these, none of the family members showed any significant differential expression when compared to the WT control cells or the reintegrant control strain (Table 2), suggesting the lack of complementation upon *PMT2* disruption.

### 3.2. The Candida albicans pmt2∆ Null Mutant Showed Defects in Cell Wall Composition and Organization 

**Figure 4:**
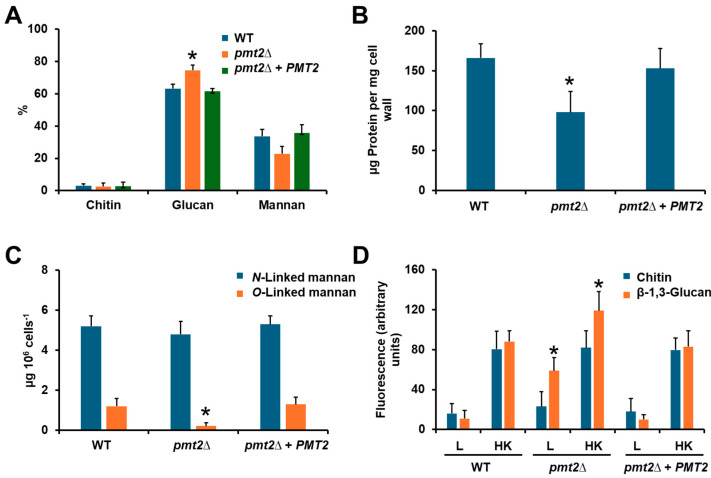
Cell wall analysis of *Candida tropicalis* wild-type, *pmt2*∆, and reintegrant control strains. In (**A**), cell walls were acid hydrolyzed and monosaccharides were analyzed by high-performance anion-exchange chromatography with pulsed amperometric detection. In (**B**), cell walls were alkali-hydrolyzed and used to quantify cell wall protein content with a colorimetric method. In (**C**), cell walls were trimmed with endoglycosidase H or β-elimination, releasing *N*-linked or *O*-linked mannans, respectively. The free glycans were quantified and data were normalized to 10^9^ yeasts. In (**D**), yeasts were incubated with fluorescein isothiocyanate conjugated wheat germ agglutinin or IgG Fc-dectin-1 chimera and anti-Fc IgG-fluorescein isothiocyanate specific lectins to label either chitin or β-1,3-glucan, respectively, and the fluorescence of 300 cells was estimated. Data are shown as mean ± SD of three independent experiments performed in duplicate. * *p* < 0.05 when compared to WT, or reintegrant control strain. Strains used are MYA-3404 (WT), HMY218 (*pmt2*Δ), and HMY220 (*pmt2*Δ + *PMT2*).

Since *O*-linked mannans are found as part of the cell wall proteins, we hypothesized that *PMT2* disruption may affect wall composition. Cell walls were isolated from yeasts, acid hydrolyzed to break down polysaccharides and oligosaccharides, and the released monosaccharides were analyzed by HPAEC-PAD [17,22]. The null mutant strain showed mannan levels similar to the WT and control cells (*p* = 0.098), but a significant increment in glucan content (Figure 4A). The chitin content was not affected by *PMT2* disruption (Figure 4A). In addition, the cell wall protein content was reduced in the null mutant, a phenotype that was restored to WT levels in the reintegrant control strain (Figure 4B). When *N*- and *O*-linked mannans were trimmed off from walls and quantified, we observed similar *N*-linked mannan levels for WT, mutant, and reintegrant control strain, but for *O*-linked mannans, these were reduced by about 75% in the *pmt2*∆ null mutant strain (Figure 4C). Once again, this wall component was found at WT levels in the reintegrant control strain (Figure 4C). Since the β-glucan levels were increased in the *pmt2*∆ null mutant strain, we also measured the relative exposure of this structural polysaccharide at the cell wall surface. We used a lectin-based approach where bulky lectins are only accessible to their targets if they are exposed to the cell surface [52,69]. Chitin was partially exposed at the cell surface of the WT strain since lectin labeling readily occurred in artificially permeabilized walls by heat (heat-killed cells, HK) than in live cells (Figure 4D). A similar profile was observed with both the mutant and reintegrant strain, suggesting no changes in chitin exposure at the cell surface (Figure 4D). The β-1,3-glucan labeling followed the same trend in the WT strain, with poor labeling in live cells and significant levels of fluorescence associated with HK cells (Figure 4D). However, the *pmt2*∆ null mutant showed higher β-1,3-glucan labeling in both live and HK cells, suggesting increased exposure on the cell surface and higher cell wall content, respectively (Figure 4D). This labeling was restored to WT levels in the reintegrant control strain (Figure 4D). The cell wall porosity and the phosphomannan content were not affected in the *pmt2*∆ null mutant (relative porosity to DEAE-dextran: 52.4 ± 6.4 and 50.6 ± 5.5 for WT and null mutant, respectively; µg of Alcian blue bound per OD_600nm_ = 1.0:90.6 ± 8.9 and 88.4 ± 6.6 for WT and *pmt2*∆ null mutant, respectively). 

Changes in cell wall components are associated with the activation of the cell wall integrity pathway [70,71,72,73,74]. This adaptation mechanism involves the activation of Pkc1 and Mkc1, leading to compensatory increments of structural polysaccharides [17,71]. Thus, the increment of β-1,3-glucan in the *C. tropicalis pmt2*∆ null mutant may be explained by the activation of the cell wall integrity pathway. The expression of both genes was upregulated in the *pmt2*∆ null mutant when compared to the WT strain (Table 3). As expected, the gene expression was restored to WT levels in the reintegrant control strain (Table 3). These data suggest activation of the cell wall integrity pathway upon *PMT2* disruption. 

The reduction of cell wall protein content in the *pmt2*∆ null mutant may reflect a defect in protein secretory pathways that deliver polypeptides to the cell wall. Thus, we measured two well-known *C. tropicalis*-secreted enzyme activities: acid proteinase, and lipase [75,76,77]. Both secreted protease and lipase activities were significantly reduced in the *pmt2*∆ null mutant when compared to the WT cells, and this phenotype was restored in the reintegrant control strain (Table 4). In agreement with a defect in protein secretion, the null mutant accumulated both activities intracellularly Thus, these results suggest that *PMT2* disruption affects secretory pathways in *C. tropicalis*.

### 3.3. The Candida tropicalis pmt2∆ Null Mutant Showed Defects in the Sensitivity to Cell Wall-Perturbing Agents, Biofilm Formation, and Adhesion

**Figure 5:**
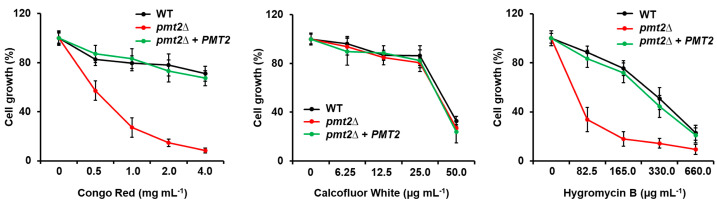
Susceptibility to Congo red, calcofluor white, and hygromycin B in the *Candida tropicalis pmt2*∆. Yeasts were incubated in YPD added with different concentrations of Congo red, calcofluor white, or hygromycin B; plates incubated for 24 h at 30 °C; and cell growth was measured by reading absorbance at 600 nm. Results are shown as a percentage of those obtained with control cultures were no perturbing agent was included. The same strain grown in the absence of any perturbing agent corresponds to 100%. Data are means ± SD of three independent experiments performed in duplicates. Strains used are MYA-3404 (WT), HMY218 (*pmt2*Δ), and HMY220 (*pmt2*Δ + *PMT2*).

Since the *C. albicans PMT2*/*pmt2*∆ mutant showed defects in the sensitivity to cell wall-perturbing agents [39], we also assessed whether this phenotype was present in the *C. tropicalis pmt2*∆ null mutant. The null mutant cells showed increased sensitivity to Congo red and hygromycin B (Figure 5), and this phenotype was restored in the reintegrant control strains (Figure 5). In line with our observations of chitin levels in the mutant cells, *PMT2* disruption did not affect sensitivity to calcofluor white (Figure 5).

The sensitivity to some antifungal drugs was also tested but the *pmt2*∆ null mutant did not show significant differences when compared to the WT cells (MIC of 0.5 µg mL^−1^ for amphotericin B for both strains; MIC of 1.0 µg mL^−1^ for caspofungin for both strains; and MIC of 4.0 µg mL^−1^ fluconazole for both strains). Regarding biofilm formation, the *pmt2*∆ null mutant showed a defect in establishing a biofilm, with a reduction in the cell content and extracellular matrix components such as glucose, glucosamine, and protein, signature elements of *C. tropicalis* biofilms [62].

**Figure 6:**
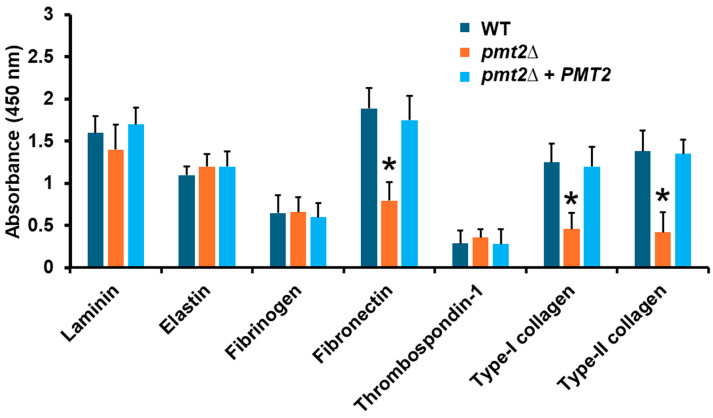
Analysis of *Candida tropicalis* adhesion to extracellular matrix components by ELISA. The extracellular matrix proteins were used to coat 96-well plates, yeasts were added to the plates, nonadherent cells were washed, and adhesion was detected with anti-*Candida* polyclonal antibodies and peroxidase-conjugated anti-rabbit IgG. Results are means ± SD of three independent experiments performed in duplicate. * *p* < 0.05 when compared to wild-type (WT) strain. Strains used are MYA-3404 (WT), HMY218 (*pmt2*Δ), and HMY220 (*pmt2*Δ + *PMT2*).

Next, the adhesive properties of the *pmt2*∆ null mutant were investigated using different components of the extracellular matrix. We used an ELISA-based approach that has been previously useful to analyze the adhesion of other fungal species with extracellular matrix components [63]. The WT control strain showed high adhesive levels to laminin, and fibronectin, intermediate levels to elastin and type I and type II collagen, and low adhesion to fibrinogen and thrombospondin-1 (Figure 6). The WT and the *pmt2*∆ null mutant showed similar adhesion levels to laminin, elastin, fibrinogen, and thrombospondin-1, but low adhesion to fibronectin and type I and type II collagen (Figure 6). The reintegrant control strain showed an adhesion profile similar to that observed in the WT strain. Collectively, these data indicate that *PMT2* disruption affects *C. tropicalis* sensitivity to cell wall-perturbing agents, biofilm formation, and adhesion to extracellular matrix components.

### 3.4. The PMT2 Disruption Affected the Candida tropicalis Interaction with Human Peripheral Blood Mononuclear Cells and Monocyte-Derived Macrophages

**Figure 7:**
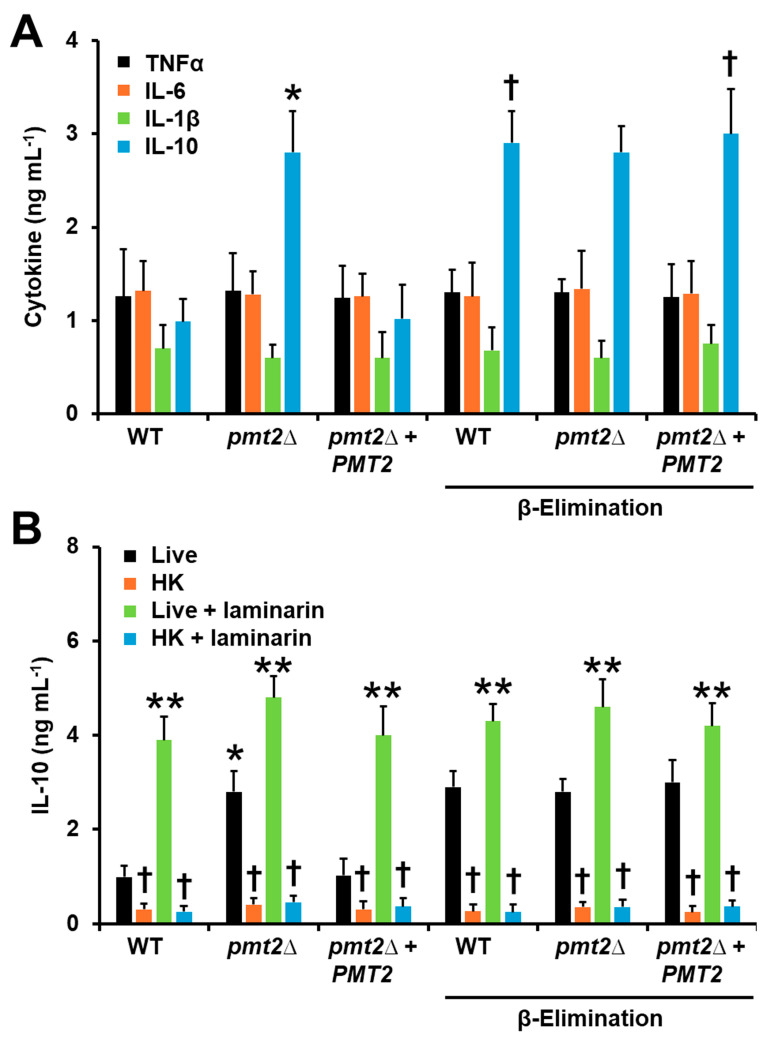
Cytokine stimulation in human peripheral blood mononuclear cells. In (**A**), yeast and human cells were co-incubated at 37 °C for 24 h, and the secreted cytokines were quantified by ELISA. * *p* < 0.05 when compared to wild-type (WT) strain. ^†^ *p* < 0.05 when compared to untreated cells. In (B), human cells were preincubated with 200 μg mL−1 laminarin before the interactions with yeasts. HK: heat-killed cells. * *p* < 0.05 when compared to WT strain. ** *p* < 0.05 when compared to live yeasts. ^†^ *p* < 0.05 when compared to untreated cells. Data are means ± SD obtained with samples from eight donors, assayed in duplicate wells. Strains used are MYA-3404 (WT), HMY218 (*pmt2*Δ), and HMY220 (*pmt2*Δ + *PMT2*).

To assess the impact of *PMT2* disruption on the ability of *C. tropicalis* to interact with the host, we first assessed ex vivo the ability to stimulate cytokine production by human PBMCs. Our group previously analyzed the cytokine profile of the WT strain and β-eliminated yeasts and found that only IL-10 production is affected, increasing the levels upon chemical elimination of *O*-linked mannan [17]. Here, the proinflammatory cytokines TNFα, IL-1β, and IL-6 were stimulated at the same level in the WT, *pmt2*∆, and reintegrant control strain, indicating a dispensable role of *O*-linked mannans for the stimulation of these cytokines (Figure 7A). However, IL-10 production was positively affected in the *pmt2*∆ null mutant strain (Figure 7A). When cells were β-eliminated, an increment only in IL-10 levels was observed in human cells stimulated with the WT and reintegrant control strains, to a similar extent as the levels stimulated by the null mutant strain (Figure 7A). The IL-10 levels stimulated by the mutant were similar to the untreated and β-eliminated cells (Figure 7A). Since IL-10 was the only affected cytokine by *PMT2* disruption, we further analyzed the stimulation of this cytokine, using heat-killed (HK) cells. This treatment artifactually exposed inner wall components at the cell surface, allowing for their interaction with immune receptors [43,78]. As previously reported, HK cells stimulated higher IL-10 levels than live cells [17] in the case of the WT and reintegrant control strain (Figure 7B). A similar trend was observed for β-eliminated cells and the *pmt2*∆ null mutant, indicating inner wall components play a dominant role in IL-10 stimulation (Figure 7B). Previously, it was demonstrated that dectin-1–β-1,3-glucan interaction is dominant in cytokine stimulation by *C. tropicalis* [17]. Thus, we hypothesize the increased ability of the *pmt2*∆ null mutant to stimulate IL-10 may be related to this pathway. Laminarin is a blocking agent for dectin-1 and precludes its interaction with its ligand [79]. Human PBMCs preincubated with laminarin were unable to stimulate high levels of IL-10 in the three studied strains, regardless they were live, HK, or β-eliminated cells, suggesting dectin-1 is involved in the increased ability of the *pmt2*∆ null mutant to stimulate this cytokine (Figure 7B).

**Figure 8:**
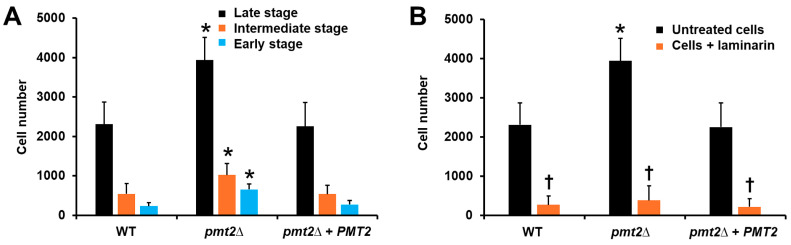
Phagocytosis of *Candida tropicalis* by human monocyte-derived macrophages. In (**A**), yeasts and human monocyte-derived macrophages were co-incubated at 37 °C for 2 h and 5% (*v*/*v*) CO_2_ and fungal uptake were analyzed by flow cytometry. Depending on the positivity in the different detection channels, human cells interacting with at least one yeast were classified as in phagocytosis’s early, intermediate, and late stages. In (**B**), similar experiments as those described in (**A**) were performed but human cells were preincubated with 200 µg mL^−1^ laminarin for 1 h at 37 °C. Here, only cells in the late stage of phagocytosis were plotted. * *p* < 0.05 when compared to wild-type (WT) cells. ^†^ *p* < 0.05 when compared to untreated cells. Results are shown as mean ± SD with samples from eight donors analyzed in duplicate. Strains used are MYA-3404 (WT), HMY218 (*pmt2*Δ), and HMY220 (*pmt2*Δ + *PMT2*).

When the mutant strain was used in phagocytosis experiments, along with human monocyte-derived macrophages, we observed increased cell numbers in the early, intermediate, and late stages of phagocytosis compared to the WT strain (Figure 8A). The reintegrant control strain showed uptake levels comparable to those observed with the WT strain (Figure 8B). The results related to cell wall composition, and IL-10 stimulation in human PBMCs, along with the observation that dectin-1 is the main player in *C. tropicalis* uptake by human monocyte-derived macrophages [17], led us to hypothesize that the increased phagocytosis in the *pmt2*∆ null mutant could be due to the interaction of dectin-1 with β-1,3-glucan. When the human cells were preincubated with laminarin, fungal uptake was significantly reduced for the three fungal strains, supporting our hypothesis. Overall, the results indicate the *PMT2* disruption affected the interaction of *C. tropicalis* with human PBMCs and monocyte-derived macrophages.

### 3.5. Candida tropicalis PMT2 Disruption Lead to Virulence Attenuation in Galleria mellonella

**Figure 9:**
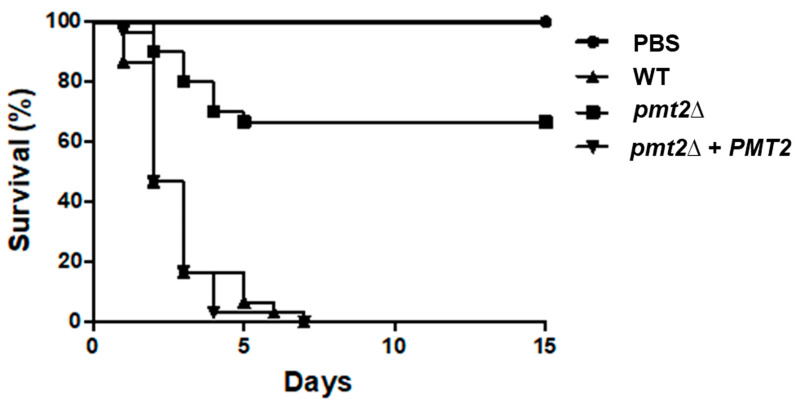
The *Candida tropicalis pmt2*∆ null mutant shows virulence attenuation in *Galleria mellonella* larvae. Larva groups, containing 30 individuals were inoculated with 2.0 × 10^7^ yeasts of the corresponding strain, and mortality was recorded daily. PBS refers to a larva group inoculated only with PBS, the vehicle used to prepare fungal inoculums. Data are shown in Kaplan–Meier plots. Strains used are MYA-3404 (WT), HMY218 (*pmt2*Δ), and HMY220 (*pmt2*Δ + *PMT2*).

Virulence was analyzed in *G. mellonella* larvae, in groups containing 30 individuals. After injection of a lethal concentration of *C. tropicalis* into the hemocele [67], the animal group inoculated with the WT strain showed a median survival of 2.0 ± 0.5 days, whereas the animal group inoculated with the *pmt2*∆ null mutant had a median survival of more than 15 days, and only 33 ± 3.0% and larvae die during the observation period (Figure 9). The reintegrant control strain showed a killing curve similar to that generated with the WT strain, with a median survival of 2.0 ± 1.0 days (Figure 9). The control group where larvae were inoculated only with PBS did not show mortality during the 15 days of observation (Figure 9). It is noteworthy that despite these differences in the killing curves, the fungal burden in the larva hemolymph was similar for the three strains (Table 5).

Previous work has proposed early virulence predictors when *C. tropicalis* interacts with *G. mellonella* larvae [67]. There are cellular and humoral immunological parameters, along with cytotoxicity that change depending on the virulence of the isolate [67]. Hemocyte content was reduced in the hemolymph of animals inoculated with WT or the reintegrant control strain but not with the *pmt2*∆ null mutant, which was closer to the levels observed in the group inoculated with PBS (Table 5). Similarly, melanin and phenoloxidase activity in hemolymph tended to increase in larvae infected with the WT or reintegrant strain, while both parameters were significantly reduced in larvae infected with the *pmt2*∆ null mutant (Table 5). Cytotoxicity, measured as the cell-free lactate dehydrogenase levels found in hemolymph [80], was high in the animal group inoculated with the WT or reintegrant control strain but lower in that interacting with the null mutant. In all cases, this animal group was closer to the values generated with the PBS-injected group than to that inoculated with the WT strain (Table 5). Collectively, these data indicate that *PMT2* is required for *C. tropicalis* virulence in *G. mellonella* larvae.

## 4. Discussion

As mentioned, *C. tropicalis* is a relevant pathogen for humans, and despite the high frequency of isolation from candidiasis and candidemia cases, little is known about basic biological aspects [81]. This may be explained by the fact that *C. albicans* is a model organism to study yeast-caused mycosis and it has frequently been assumed that observations in this organism may be extrapolated to other members of the same genus. However, there is growing evidence that these organisms have significant genomic and phenotypical differences [81,82,83,84,85]. Thus, it is not surprising that in this case, the relevance of *PMT2* for cell viability in *C. tropicalis* is different from that observed in *C. albicans*. In *S. cerevisiae*, *PMT2* disruption is not lethal [86], but in *Cryptococcus neoformans*, it is an essential gene [87], suggesting that the essentiality of this gene might be a species-specific trait. We currently do not have an explanation based on experimental data for the discrepant role of *PMT2* in these two *Candida* species. It is known that pmt2 has interactions with pmt1 and pmt5 in *S. cerevisiae* [88]. These interactions between different family members determine the targeted protein to be glycosylated, including proteins for cell division, growth, mating, and cell wall fitness [37]. It is assumed that similar interactions would occur in *C. albicans* [89], even though no experimental proof has been reported. Thus, it is possible to speculate that these protein–protein interactions may occur differently in these fungal species, and consequently, the target proteins for Pmt2 or the dimers including this protein are different. This implies that in *C. tropicalis*, the Pmt2 targets are proteins involved in non-essential processes. This is in line with the observation that cells did not aggregate and showed a canonical morphology. One possible explanation for the defect in growth rate and filamentation is associated with the observed defects in the cell wall. These phenotypes have been reported in *C. albicans* and *C. tropicalis* null mutants with defects in the cell wall composition or organization [17,19,20,21,22,39,46,52].

We have previously reported that *O*-linked mannans are a minor component of the *C. tropicalis* cell wall [17], and our results here confirm this observation. Even though there was a significant reduction in *O*-linked mannan content, the total cell wall mannan levels were not significantly affected. The transcriptional levels of *C. albicans PMT1*, *PMT2*, and *PMT4* are similar under normal growth conditions [90], and this is different in *C. tropicalis*, where *PMT2* and *PMT4* are expressed more than *PMT1*. This may explain the drastic reduction in *O*-linked mannans in the null mutant and also suggest that pmt2 may work in collaboration with Pmt4. Previously, the *C. albicans* heterozygous *PMT2*/*pmt2*∆ null mutant did not show changes in the cell wall composition [39], contrasting with the increment in β-1,3-glucan content, its exposure at the cell surface, and the activation of the cell wall integrity pathway in the *C. tropicalis* mutant. Once again, this difference may be explained by different target proteins for Pmt2 in the two species. In line with this hypothesis, in *S. cerevisiae*, there are target proteins for Pmt2 that are involved in cell wall elaboration and integrity [37].

The contribution of *O*-linked mannosylation for proper biofilm formation was analyzed in *C. albicans*, and Pmt1, Pmt2, Pmt4, and Pmt6 are required for the establishment and maturation of biofilms [91]. Here, we found a similar observation for *C. tropicalis PMT2,* indicating a conserved contribution of this protein for biofilm formation in both species. It was previously demonstrated that *N*-linked mannosylation is relevant for biofilm formation in *C. tropicalis*, as disruption of this metabolic pathway negatively affected protein secretion and biofilm maturation [36]. Because both biofilm formation and protein secretion were negatively affected by *PMT2* disruption, it is feasible to propose a similar role for *O*-linked mannosylation in biofilm formation. 

*C. tropicalis* can bind different types of primary human cells, cell lines, and to the surface of indwelling materials [84]. Thus far, little is known about the ability of this organism to adhere to extracellular matrix components. It can bind fibronectin, vitronectin, and laminin, with the binding to fibronectin being sensitive to cell pretreatment with mannosidase [92]. This mannosidase treatment is expected to trim mannans from cell wall glycoproteins and, consequently, disrupt its structure and function [92]. The reduction in binding to fibronectin by the *pmt2*∆ null mutant is in line with this observation and indicates that glycoproteins containing *O*-linked mannans are responsible for the binding of this extracellular matrix component. No evidence in the literature was found regarding the binding to collagens, making this report the first to document this observation. In *C. albicans*, the heterozygous *PMT2*/*pmt2*∆ null mutant did not show adhesion defects to an oral epithelial cell line, but the double mutant *mnt1*-*mnt2*∆ null mutant, which has a defect in the elongation of the *O*-linked mannans [23], showed lower adhesion to buccal epithelial cells, indicating that *O*-linked mannans in both organisms have similar relevance for adhesion.

The role of *O*-linked mannans during the interaction of *C. albicans* or *C. tropicalis* with human PBMCs is minor during cytokine stimulation [17,26,27]. The loss of elongated *O*-linked mannans marginally affects the stimulation of proinflammatory cytokines, and thus far, only IL-10 levels are affected in *C. tropicalis* with truncated *O*-linked mannans on the cell surface [17,27]. Here, our results showed a similar trend for the *pmt2*∆ null mutant, confirming these observations and offering an explanation for the incremented levels of Il-10, namely, increased engagement of dectin-1 with β-1,3-glucan exposed at the cell surface. Similar to this observation, it was reported that the *C. albicans* heterozygous *PMT2*/*pmt2*∆ null mutant stimulated increased IL-10 levels in murine macrophages [93]. Our phagocytosis results are in line with observations in *C. albicans*. The *mnt1*-*mnt2*∆ null mutant showed increased phagocytosis by murine macrophages, similar to the *C. tropicalis pmt2*∆ null mutant. However, the phagocytosis event is different in both species, as β-1,3-glucan and *N*-linked mannans are the main players of the uptake event in *C. albicans*, while phosphomannan and β-1,3-glucan are the main cell wall components involved in *C. tropicalis* phagocytosis [17,24,94].

The *PMT* family is required for virulence in *C. albicans*, and the heterozygous *PMT2*/*pmt2*∆ null mutant showed virulence attenuation in a murine model of systemic candidiasis [95], and defects in invasion and damage to an oral epithelial cell line [96]. Here, the *C. tropicalis pmt2*∆ null mutant also showed an avirulent phenotype, indicating this role is conserved in both species. Despite this observation, it is unlikely this protein is a virulence factor, because it has no direct function in host damage. Instead, it may be considered a virulence determinant. Similar CFUs recovered from larvae suggest that the growth defect observed in vitro is not occurring in the in vivo setting. This implies that the relevance of this gene for cell growth and adaptation to different growing conditions varies depending on the environment. This is supported by our results about the *PMT2* expression levels under different growing conditions. Similarly, it was reported that the behavior of the *C. albicans* heterozygous *PMT2*/*pmt2*∆ null mutant, in terms of filamentation, changes in vitro and in vivo conditions [39,93].

In conclusion, we report here that a *C. tropicalis pmt2*∆ null mutant was generated, and its phenotypical characterization indicated this gene is required for proper cell wall organization, biofilm formation, adhesion to fibronectin, and type I and type II collagen. In addition, the *PMT2* loss led to defects in the interaction with human PBMCs, monocyte-derived macrophages, and virulence attenuation in *G. mellonella*. 

## Figures and Tables

**Table 2:** Analysis of the *Candida tropicalis PMT* family member expression in different growing conditions.

Strain	*PMT1*	*PMT2*	*PMT4*	*PMT5*	*PMT6*
YPD ^a^					
Wild-type	1.0 ± 0.2	3.3 ± 0.4	2.4 ± 0.6	0.8 ± 0.3	0.6 ± 0.3
*pmt2*∆	0.9 ± 0.4	N.D. ^d^	2.8 ± 0.3	0.9 ± 0.5	0.5 ± 0.4
*pmt2*∆ + *PMT2*	0.9 ± 0.3	3.1 ± 0.3	2.6 ± 0.7	0.6 ± 0.4	0.5 ± 0.4
SC ^b^					
Wild-type	1.6 ± 0.4	2.9 ± 0.4	3.9 ± 0.7	0.9 ± 0.5	1.1 ± 0.5
*pmt2*∆	1.8 ± 0.3	N.D.	4.2 ± 0.8	1.1 ± 0.5	0.8 ± 0.4
*pmt2*∆ + *PMT2*	1.8 ± 1.6	2.6 ± 0.5	4.0 ± 0.9	1.0 ± 0.6	0.9 ± 0.5
RPMI + FCS ^c^					
Wild-type	2.4 ± 0.4	5.2 ± 0.6	5.2 ± 0.9	1.1 ± 0.2	3.1 ± 0.4
*pmt2*∆	2.6 ± 0.5	N.D.	4.9 ± 0.8	1.4 ± 0.4	3.5 ± 0.6
*pmt2*∆ + *PMT2*	2.2 ± 0.5	5.0 ± 0.5	5.3 ± 1.1	1.0± 0.5	3.0 ± 0.5

The *ACT1* gene was used for data normalization. Data are means ± SD of three independent experiments performed in duplicate. ^a^ Cells were grown at 28 °C for 24 h. ^b^ Cells were grown at 28 °C for 48 h. ^c^ Cells were grown for 3.5 h at 37 °C. ^d^ Not detected.

**Table 3:** Gene expression analysis and secreted enzyme activity in *Candida tropicalis* wild-type, *pmt2*∆, and reintegrant control strains.

Strain	*PKC1* ^a^	*MKC1* ^a^	Secreted Protease (U) ^b^	Secreted Lipase (U) ^c^
Wild-type	1.2 ± 0.5	1.0 ± 0.3	1122.4 ± 223.4	395.5 ± 66.5
*pmt2*∆	3.8 ± 0.8 *	4.6 ± 0.6 *	589.5 ± 179.6 *	99.2 ± 49.7 *
*pmt2*∆ + *PMT2*	1.0 ± 0.4	1.3 ± 0.5	1098.2 ± 186.4	402.5± 63.0

^a^ The *ACT1* gene was used for data normalization. ^b^ Units defined as the change in absorbance at 280 nm per minute. ^c^ Units defined as nmoles 4-methylumbelliferone generated per minute. Data are means ± SD of three independent experiments performed in duplicate. * *p* < 0.05 when compared to wild-type or *pmt2*∆ + *PMT2* strains.

**Table 4:** Biofilm formation and analysis of the extracellular matrix in *Candida tropicalis* wild-type, *pmt2*∆, and reintegrant control strains.

Strain	Biofilm Formation ^a^	CFU (×10^7^ cells) ^b^	Glucose (µg mL^−1^) ^c^	Glucosamine (µg mL^−1^) ^c^	Protein (µg mL^−1^) ^c^
Wild-type	4.1 ± 0.2	1.4 ± 0.6	40.4 ± 6.2	563.8 ± 45.6	65.5 ± 9.8
*pmt2*∆	2.7 ± 0.2 *	0.9 ± 0.2 *	18.5 ± 5.6 *	265.6 ± 33.5 *	22.8 ± 15.6 *
*pmt2*∆ + *PMT2*	4.0 ± 0.3	1.5 ± 0.3	37.7 ± 86.4	532.6 ± 33.8	61.5 ± 11.6

^a^ Biofilm formation was assessed by measuring crystal violet reduction at 590 nm. ^b^ Colony-forming units (CFUs) were calculated after biofilms were incubated with chitinase for cell detachment from wells. ^c^ Quantified from cell-free biofilm preparations obtained with chitinase. Data are means ± SD of three independent experiments performed in duplicate. * *p* < 0.05 when compared to wild-type or *pmt2*∆ + *PMT2* strains.

**Table 5:** Colony-forming units, cytotoxicity, hemocyte, melanin, and phenoloxidase levels in *Galleria mellonella* larvae inoculated with *Candida tropicalis*.

Strain	CFUs (×10^7^) ^a^	Cytotoxicity (%) ^b^	Hemocytes (×10^6^) mL^−1^	Melanin ^c^	Phenoloxidase ^d^
PBS ^e^	0.0 ± 0.0	9.8 ± 4.2	5.4 ± 0.4	1.1 ± 0.2	0.3 ± 0.1
WT	1.3 ± 0.4	78.9 ± 9.6	2.2 ± 0.5	4.2 ± 0.4	2.9 ± 0.3
*pmt2* ∆	1.4 ± 0.3	26.4 ± 11.5 *	4.9 ± 0.2 *	2.3 ± 0.5 *	1.1 ± 0.2 *
*pmt2*∆ + *PMT2*	1.3 ± 0.5	81.2 ± 15.4	2.5 ± 0.6	4.4 ± 0.2	3.2 ± 0.6

^a^ Hemolymph was collected from decapitated larvae and used to quantify the colony-forming units (CFUs) by serial dilutions in YPD plates. ^b^ Lactate dehydrogenase activity was quantified in cell-free hemolymph. Data are normalized to a positive control (lysed hemocytes) that corresponds to 100% activity. ^c^ Measured in the cell-free hemolymph at A_405nm_. ^d^ Phenoloxidase activity defined as the Δ_490nm_ min^−1^ μg protein ^−1^. ^e^ Larvae inoculated only with PBS. Strains used are MYA-3404 (WT), HMY218 (*pmt2*Δ), and HMY220 (*pmt2*Δ + *PMT2*). * *p* < 0.05 when compared with WT strain.

## Data Availability

The original contributions presented in the study are included in the article, further inquiries can be directed to the corresponding author.
